# The Alterations of Biofilm Formation and EPS Characteristics of a Diatom by a Sponge-Associated Bacterium *Psychrobacter* sp.

**DOI:** 10.1155/2018/1892520

**Published:** 2018-06-24

**Authors:** Xiaojian Zhou, Jie Meng, Zhaowei Yu, Li Miao, Cuili Jin

**Affiliations:** ^1^College of Environmental Science and Engineering, Yangzhou University, No. 196 Huayang West Street, Hanjiang District, Yangzhou, Jiangsu, China; ^2^Marine Science and Technology Institute, Yangzhou University, No. 196 Huayang West Street, Hanjiang District, Yangzhou, Jiangsu, China

## Abstract

A sponge-associated bacterium, which was identified as *Psychrobacter* sp. in this study, was found with high activity against biofilm formation of benthic diatoms, including *Amphora* sp., *Nitzschia closterium*, *Nitzschia frustulum*, and *Stauroneis* sp. The activity against diatom biofilm formation by the tested strain was confirmed mostly in the culture supernatant and could be extracted using organic solvents. Treatment with its supernatant crude extract significantly reduced the cells of *Stauroneis* sp. forming biofilm and slightly increased the cells floating in the culture medium, which results in the ratio of biofilm cell/floating cell altering from 0.736 in control to 0.414 in treatment. Use of the supernatant crude extract led to increased production of extracellular polymeric substances (EPSs) by diatom *Stauroneis* sp. from 16.66 to 41.59 (g/g cell dry weight). The increase in EPS production was mainly contributed by soluble EPS (SL-EPS) and followed by the EPS that was tightly bound to biofilm cells (BF-TB-EPS). In addition, the supernatant crude extract caused significant changes in the monosaccharides composition of the EPS of *Stauroneis* sp. Specifically, glucuronic acid (Glc-A) and *N*-acetyl-D-glucosamine (Glc-NAc) in BF-TB-EPS were 55% fold decreased and 1219% fold increased, respectively. Based on our findings, we proposed that these changes in monosaccharides composition might lead to a decreased biofilm formation efficiency of diatom.

## 1. Introduction

Biofouling of ship hulls by biofilms of algae and bacteria and by larger organisms, for example, barnacles and mussels (macrofouling), causes large economic losses worldwide [1]. In addition to increased drag and decreased speed by 8–21% of ships, biofilms on ship hulls also contribute for inducing subsequent macrofouling [2]. Every surface that is immersed in seawater becomes rapidly covered with an unavoidable biofilm. However, biofilm formation is a complex multistage process and not yet thoroughly investigated [3]. The microbial communities of biofilms are commonly dominated by diatoms in light-penetrating marine habitats [4–6]. Benthic diatoms, which are capable of forming biofilm even on the most fouling resistant surfaces, play a key role in the biofilm development [7]. Therefore, interruption of diatom biofilm formation is an essential and challenging step for resolving the biofouling problem [8, 9].

Photoautotrophic biofilms are composed of cells that are embedded in extracellular polymeric substances (EPSs) [10]. Marine benthic diatoms secrete large amounts of EPSs into the surrounding environment, as much as approximately 30–60% of photoassimilated carbon [11, 12]. These EPSs are mainly composed of carbohydrates and proteins and play various roles in the life of diatoms. EPS can act as a diffusive barrier against chemicals, physical stress, dehydration, and predator grazing. EPS can also cause the retention of exoenzymes or cellular metabolites and nutrient sequestration from aquatic environments. EPS also promotes sorption of compounds, cell-to-cell communication, and formation of microcolonies [11, 13, 14]. Besides, EPS acts as a type of glue that is primarily used by benthic diatoms for aggregation and gripping to substrates and is also involved in the motility system and substratum adhesion of diatoms [15–17]. Therefore, EPS is proposed to be a key component for diatom cells to form biofilms on the substratum beneath water [16].

In marine environments, sponges are commonly fouling-free due to their chemical defenses against fouling organisms [18, 19]. Many natural compounds were isolated from sponges and found to be similar to those from microorganisms. Moreover, some of them were verified being microbially produced by sponge-associated microorganisms [20]. Strong antimicrobial and antifouling activities, including inhibition against the biofilm formation and larval settlement of typical fouling organisms such as *Hydroides elegans* or *Amphibalanus amphitrite,* were found among the metabolites of sponge-associated bacteria [9, 18, 19, 21–28]. However, few study on how the metabolites of active bacterial strains affect diatom biofilm formation and whether treatment with these metabolites provokes changes in diatom EPS is reported. Currently, the active natural products from sponge-associated bacteria against diatom biofilm formation have not been sufficiently investigated yet [29].

Our previous studies highlighted the strong activities against biofilm formation of several diatom species by crude extracts from the whole culture of some sponge-associated bacteria [29, 30]. Among the tested diatoms, the benthic diatom *Stauroneis* sp. was used as a model organism in this study because its gliding mechanism and monosaccharide composition of the EPS are very clear [31, 32]. In the present study, we found another active sponge-associated strain and would like to clarify several questions: (1) what portion of crude extracts from active bacterial culture is responsible for the activity against diatom biofilm formation, (2) whether the active strain inhibits diatom biofilm formation by altering cell distribution in biofilm and planktonic phase rather than by reducing total cell biomass in the whole culture, and (3) whether correlating responses in the EPS production or composition occur with the poor biofilm formation efficiency of diatoms when treated by the active strain.

## 2. Materials and Methods

### 2.1. Bacterial Culture

Sponge-associated bacteria were originally collected from San Juan Island, Washington. Collection, isolation, and purification of the strains were performed by Coastal Marine Laboratory in Hong Kong University of Science and Technology (HKUST) as described previously [33]. One strain of UST050418-708 was selected for this study since we found that it can be active against diatom biofilm formation [29].

A stock culture of strain UST050418-708 was stored in the Marine Science and Technology Institute, Yangzhou University. The stock solution (1 ml) was inoculated in 10 ml of the peptone-yeast extract medium (P-Y medium, containing 0.3% yeast extract and 0.5% peptone in artificial seawater (ASW)) and incubated for about two days to the exponential phase at 23°C with shaking at 120 rpm. The bacterial culture in the exponential phase was then inoculated in 500 ml of the P-Y medium and incubated for three days in the stationary phase under the same conditions and used for extract preparation [29].

### 2.2. Strain Identification

The genomic DNA of strain UST050418-708 was extracted, and its 16S rDNA was amplified and sequenced by Sangon Biotech (Shanghai) Co., Ltd. as described by Jin et al. [29]. The obtained sequence was submitted to GenBank. The strain was identified by comparing the submitted sequence with those available in GenBank databases. Similar sequences were aligned using multiple sequence alignment program MEGA. Gaps and positions with ambiguities were excluded from the phylogenetic analysis. Phylogenetic analysis was performed using the neighbour-joining method described by Li et al. [34].

### 2.3. Extract Preparation from Bacterial Culture

The crude extract was collected from the bacterial culture as described by Jin et al. [29]. In detail, the extraction solvent of ethyl acetate (EA) : acetone = 95 : 5 (v/v) was added with a ratio of extraction solvent : bacterial culture = 1 : 1 (v/v) and shaken vigorously for 1 h. After standing and stratification, the organic phase was separated and dried on a rotary evaporator (37°C) to obtain the whole culture crude extract. For the preparation of supernatant crude extract, the bacterial cells were separated from the culture medium by centrifugation (5,880 ×g, 20 min, 15°C), and the supernatant was then extracted with the same extraction solvent and procedure as above. Each crude extract was dissolved in dimethylsulfoxide (DMSO) for subsequent bioassays.

### 2.4. Diatom Culture

Four benthic diatoms, *Amphora* sp., *Nitzschia closterium*, *Nitzschia frustulum*, and *Stauroneis* sp., were obtained from the Key Laboratory of Mariculture, Ministry of Education, Ocean University of China.

The diatoms were cultured by the method of Jin et al. [29]. In detail, ASW-based Guillard's *f*/2 culture medium [35] was used, and the culture conditions of 23°C, 100 *μ*mol photons m^−2^·s^−1^ illumination, and light : dark = 12 h : 12 h cycle were employed. Diatom films collected prior to assays were suspended and washed with ASW twice, adjusted to approximately 1 × 10^5^ cells·ml^−1^ in ASW, and used in following diatom biofilm formation assays.

### 2.5. Diatom Biofilm Formation Assays Using Crude Extracts

Diatom biofilm formation assays for crude extracts were performed in 24-well polystyrene plates (353047, Becton Dickinson Labware) following the method described by Jin et al. [29]. Algal suspension in ASW (1 ml) and the crude extract dissolved in DMSO (50 *μ*l) were added to each well on the plate to achieve the final concentration of 100 *μ*g·ml^−1^ for the crude extract in the well. For the control wells, 1 ml of the algal suspension in ASW and 50 *μ*l DMSO were included. After 24 h incubation at the above diatom culture conditions, the floating cells were removed by pipetting and the biofilm cells were then counted using an inverted microscope (Nikon ECLIPSE TS 100). Triplicates were done for each treatment and control. The inhibition ratios (*R*) were expressed in terms of the percentages for each sample: *R* (%) = 1 − (biofilm cell number of treatment)/(mean biofilm cell number of controls) [29, 36].

### 2.6. Diatom Biofilm Formation Assays Using Various Portions of the Bacterial Culture

**Figure 1:**
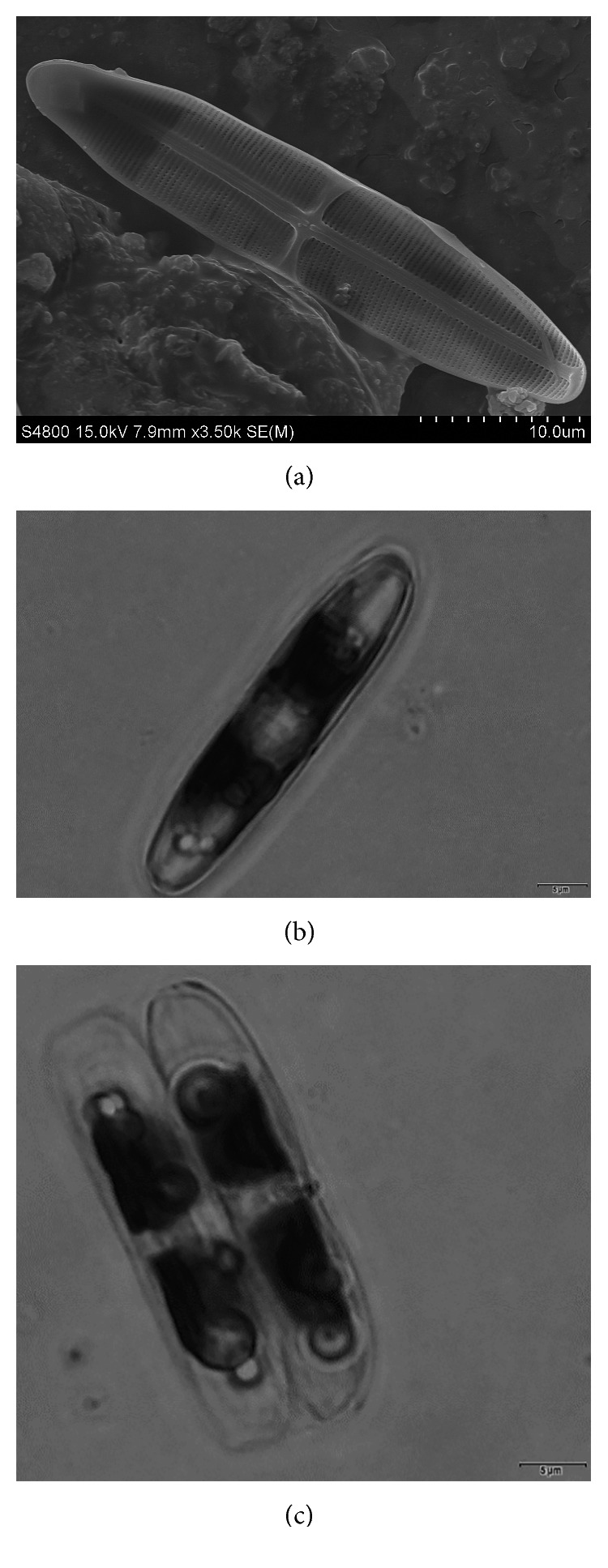
Scanning electron microscope (a) and optical microscope ((b) for valve view and (c) for girdle view) images of diatom *Stauroneis* sp.

To examine the possible trophic competition between the living bacterial cells and diatoms, the benthic diatom *Stauroneis* sp. was used in this experiment. Diatom *Stauroneis* sp. under optical and electron microscopes is shown in Figure 1. Prior to the bioassays, the densities of the suspended *Stauroneis* sp. were adjusted using a hemocytometer to 1 × 10^5^ cells·ml^−1^. The bacterial isolates were collected from stationary-phase cultures after 72 h incubations in the P-Y medium at 23°C and 120 rpm. The collected bacterial culture was centrifuged (20 min, 5,880 ×g, and 15°C). The supernatant was collected and used as the “supernatant” for the bioassay. The pellet was resuspended in the same volume of the fresh P-Y medium and used as “cells” for the bioassay.

Assays were conducted in 24-well polystyrene plates (353047, Becton Dickinson Labware) using two volume ratios (bacteria : diatom volume ratios of 3 : 7 and 5 : 5). When the ratio was 3 : 7, each well contained 0.7 ml of algal suspension in the *f*/2 medium and 0.3 ml of bacterial aliquots of the supernatant or cells. When the ratio was 5 : 5, the mixture in each well was composed of 0.5 ml of the algal suspension and 0.5 ml of bacterial aliquots. The total volume of each well for both ratios was 1 ml, and controls contained 0.3 or 0.5 ml of the sterilized P-Y medium instead of bacterial aliquots. Plates were incubated for 24 h at the same diatom culture conditions mentioned above. The contents in the wells were washed by gently pipetting, algal cells still attached to the bottom of the wells were counted, and the inhibition ratios (*R*) were calculated following the method described above [29, 36].

### 2.7. Treatment of Growing Diatom Using Bacterial Supernatant Crude Extract

The benthic diatom *Stauroneis* sp. was used in this experiment. The diatom was cultured in 2-L conical flasks. Each flask contained 1500 ml of freshly inoculated *Stauroneis* sp. with a cell density of 1 × 10^5^ cells·ml^−1^ in the sterilized *f*/2 medium. Bacterial supernatant crude extract dissolved in DMSO was added to a final concentration of 150 *μ*g·ml^−1^ at a DMSO content of 0.5% (v/v). The control included 0.5% (v/v) DMSO only. The triplicated flasks for both treatment and control were incubated for 39 days at 23°C, with 100 *μ*mol photons m^−2^·s^−1^ and a 12 h : 12 h light : dark cycle.

### 2.8. EPS Preparation from Diatoms Grown in the Presence of Bacterial Supernatant Crude Extract

The EPS fractionation procedures for the suspension fraction (cells floating in the media) and biofilm fraction (cells embedded in biofilm attached to the flask bottom) were performed as described by Xu et al. [37]. After completing the incubation, all flasks were shaken on a shaker at 70 rpm for 10 min. The suspension fraction was carefully decanted and collected after shaking. Then, ASW was added to the flask, and diatom cells in the biofilm were carefully scratched down and suspended. The detailed methods for the EPS fractionation, cell density detection, and cell dry weight measurement were as follows.

The suspension fraction of diatom culture was counted for cell density after shaking using a hemocytometer under microscopy and then centrifuged at 1,707 ×g for 20 min to separate diatom cells and the supernatant. The supernatant of suspension fraction was collected to measure the soluble EPS (SL-EPS), that is, the EPS fraction that could be removed by soft perturbation. The pellets were resuspended in ASW and heated at 40°C overnight followed by three washes with ASW and centrifugation at 1,707 ×g for 20 min. The ASW washes were collected to measure the EPS tightly bound to floating cells (F-TB-EPS), and the final pellets were placed in a 100°C oven and heated to constant weight to measure the mass of floating cells.

For the biofilm fraction of diatom culture, cells were suspended in the same volume of ASW as the original culture by vigorous shaking, and counted for cell density using a hemocytometer under microscopy. After being centrifuged at 1,707 ×g for 20 min, the supernatant was collected to measure the EPS loosely bound to the biofilm cells (BF-LB-EPS). And the pellets were treated in the same way as that for F-TB-EPS, and the supernatant was collected to measure EPS that tightly bound to biofilm cells (BF-TB-EPS). The final pellets were heated at 100°C until constant weight to measure the weight of biofilm cells.

All the EPS fraction samples were precipitated with 3-fold volumes of ethanol. The solution was left overnight in the refrigerator (4°C) [38]. The final precipitate was collected by centrifugation and washed three times with 2-fold volumes of acetone and dichloromethane subsequently to obtain the crude EPS. The crude EPS was dried under a stream of nitrogen gas, weighed, and stored at −20°C. After deproteination using the Sevag method and desalting using dialysis (3.5 kDa), the purified EPS was obtained by rotary evaporation and freeze-drying for determination of monosaccharide compositions [39].

### 2.9. Determination of Monosaccharide Compositions in EPS

The monosaccharide compositions of EPSs were determined using high-performance liquid chromatography (HPLC) after derivatization with 1-phenyl-3-methyl-5-pyrazolone (PMP) [40]. As standards, 11 monosaccharides were used: mannose (Man), glucuronic acid (Glc-A), *N*-acetyl-D-glucosamine (Glc-NAc), xylose (Xyl), galactose (Gal), arabinose (Ara), fucose (Fuc), glucose (Glc), galacturonic acid (Gal-A), rhamnose (Rha), and glucosamine hydrochloride (GlcN). The HPLC system (L2000, Hitachi, Japan) was equipped with a diode array detector (DAD, L-2455, Hitachi, Japan) installed in tandem at the outlet of the column (LaChrom, ODS C_18_, 5 *µ*m, 4.6 mm × 250 mm, Hitachi, Japan) and mounted with an ODS precolumn. The used solvents were 83% methanol and a 17% potassium dihydrogen phosphate-sodium hydroxide buffer solution (0.1 M, pH 8.6) at a fixed flow rate of 0.7 ml·min^−1^ at 25°C. A volume of 10 *µ*l of the sample was injected into the column using an autosampler (L-2200, Hitachi, Japan), and the UV absorption at *λ* = 245 nm was detected. The HPLC analyses were performed at least twice for each sample.

The data were analysed by the method of Yang et al. [41] to determine the monosaccharide ratio of each sample. The correction factors (*f*_1/2_) and molar ratios (*R*_1/2_) between every two monosaccharides ((1) and (2)) were calculated using the following equations, respectively:

(1) $$\begin{array}{c} f_{1/2}=\frac{\left(A_{2}/m_{2}\right)}{\left(A_{1}/m_{1}\right)}, \end{array}$$

(2) $$\begin{array}{c} R_{1/2}=f_{1/2}\;*\;\left(\frac{A_{1}^{\prime }}{A_{2}^{\prime }}\right), \end{array}$$

where *A*_1_ and *A*_2_ and *m*_1_ and *m*_2_ are the peak area and weight for two component monosaccharides in the standard solution, respectively, and *A*_1_′ and *A*_2_′ are the peak areas for the component monosaccharide of the tested samples [41].

The content of one of the identified monosaccharides (X) is set as 1, and the mole contents of other monosaccharides were calculated based on *f*_1/2_ and *R*_1/2_ between X and each of others. The mole percentage of each monosaccharide was calculated as its mole content divided by the sum of the mole contents of all identified monosaccharides.

For diatoms treated by the supernatant crude extract of the active strain, the amplitude of variation (%) for each monosaccharide in an EPS fraction was calculated as the variation (mole percentages) between the treatment and control divided by the mole percentage in the control.

### 2.10. Statistical Analysis

All calculations were performed with at least triplicate samples. Statistical analyses were carried out using the IBM SPSS statistics 22. The differences among treatments in each experiment were compared using the independent *t*-test or one-way analyses of variance (ANOVA) followed by the LSD test, with a threshold for significance of 0.01.

## 3. Results

### 3.1. Identification of Active Strain

**Figure 2:**
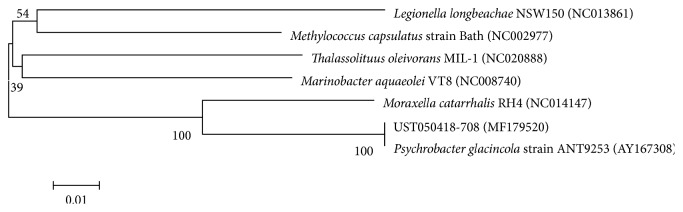
Phylogenetic tree based on 16S rDNA of strain UST050418-708. The evolutionary history was inferred using the neighbour-joining method. The numbers at the nodes indicate the bootstrap values based on neighbour-joining analyses of 1000 sample data sets. The tree is drawn to scale, with branch lengths in the same units as those of the evolutionary distances used to infer the phylogenetic tree. The evolutionary distances were computed using the Maximum Composite Likelihood method and are in the units of the number of base substitutions per site. Bar of 0.01 represents per nucleotide position. The numbers in parentheses are accession number of sequences. Evolutionary analyses were conducted in the MEGA6 software package.

Genomic DNA of strain UST050418-708 was extracted, and the 16S rDNA was PCR amplified and sequenced. The nearly complete 16S rRNA gene sequence of strain UST050418-708 (1431 bp) was obtained and submitted to GenBank with an accession number (MF179520). Comparative analysis of the 16S rRNA gene sequence with sequences deposited in GenBank using BLAST showed that the strain belong to the genus *Psychrobacter* and has a very high similarity (100%) with *Psychrobacter glacincola* (Figure 2). Therefore, the strain was identified as *Psychrobacter* sp. based on the 16S rDNA sequence.

### 3.2. The Activity against Diatom Biofilm Formation by Crude Extract from Whole Bacterial Culture

**Figure 3:**
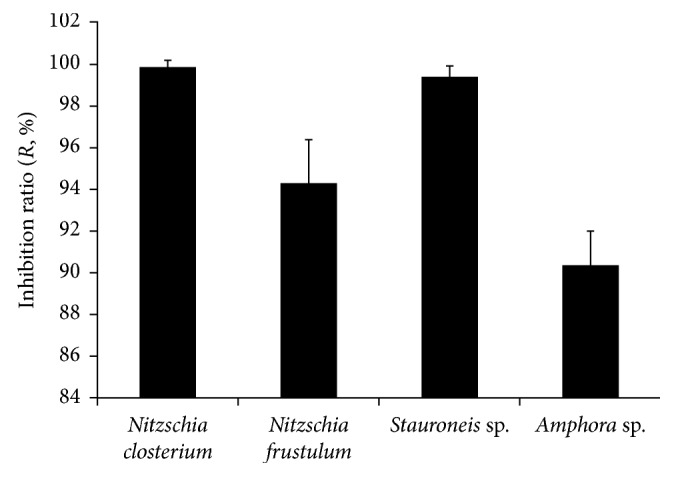
Activities against diatom biofilm formation by the crude extract from the whole culture of *Psychrobacter* sp. The inhibiting ratios (*R*) were calculated after 24 h incubations. Triplicates were tested for each treatment and control, and the means and standard deviations are shown as closed columns and bars, respectively.

A crude extract from whole culture of the tested strain was prepared and used in the diatom attachment assays. At concentration of 100 *μ*g·ml^−1^, the crude extract from the whole culture had very high activities against diatom biofilm formation and inhibited four diatom species from attaching to the bottom of 24-well plates with inhibition ratios (*R*) higher than 90% (Figure 3). For *N. closterium* and *Stauroneis* sp., the inhibition ratios were over 98%.

### 3.3. The Activity against Diatom Biofilm Formation by Various Portions of the Bacterial Culture

**Figure 4:**
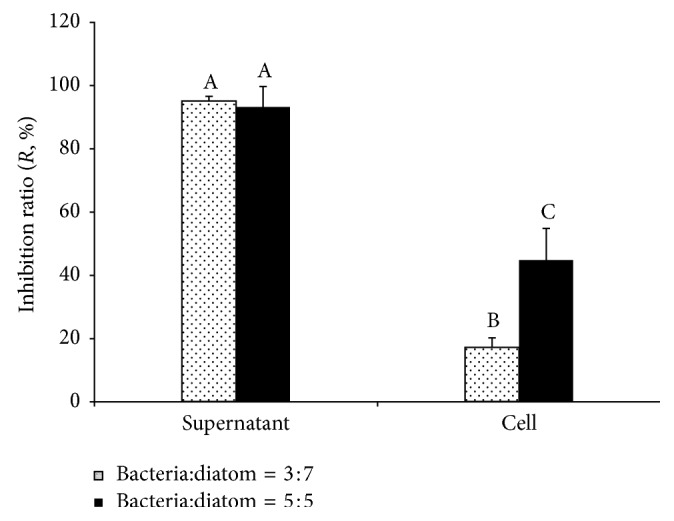
Activities against diatom biofilm formation by bacterium *Psychrobacter* sp. with different portions. The numbers of 7 : 3 or 5 : 5 indicate the volume ratios with which diatom *Stauroneis* sp. in suspension was mixed with the supernatant or cells suspension in the fresh culture medium. The inhibiting ratios (*R*) were calculated after 24 h incubations. Triplicates were tested for each treatment and control, and the means and standard deviations are shown as columns and bars, respectively. Significance was tested for each treatment separately. Same/different letters above the bars indicate no/a statistical difference in determination by one-way analyses of variance (ANOVA) followed by LSD test (*P* < 0.01), respectively.

Since the whole culture extract of tested strain inhibited diatom biofilm formation with very high efficiencies, the supernatant and cells from the bacterial culture (without extraction) were used in diatom biofilm formation assays. Regardless of the used ratios between bacteria and diatom being 3 : 7 or 5 : 5, both two fractions of the bacterial culture showed activities against diatom biofilm formation (Figure 4). Among all treatments, the supernatant showed the highest activity against diatom biofilm formation with *R* = 95%, when the ratio between bacteria and algae was 3 : 7 (Figure 4). When the ratio was adjusted to 5 : 5, the supernatant fraction maintained high activity with *R* = 93%. Thus, the supernatant of the tested strain possessed higher activity against diatom biofilm formation than the cells. Statistical analysis indicated that the supernatant showed significantly higher activity than cells, and that the change in the ratio (bacteria: diatom) significantly affected the activities of the cells rather than the supernatant.

### 3.4. Effect of the Bacterial Supernatant Crude Extract on the Growth and Biofilm Formation of Diatom

Since the supernatant was the most effective portion of the bacterial culture, a supernatant crude extract was prepared and used to treat the growing diatom of *Stauroneis* sp. The total weight of dry cells and cell density for the whole culture of treated diatom showed minor decrease from 0.578 g to 0.504 g (by percentage of 12.8%) and from 9.557 × 10^5^ ml^−1^ to 8.409 × 10^5^ ml^−1^ (by percentage of 12.0%), respectively (Table 1). Importantly, the dry cell weight in the biofilm fraction significantly decreased from 0.245 g (control) to 0.147 g (treatment) with a decrease percentage of 40.0%. At the same time, the dry cell weight in the suspension fraction increased slightly with 7.2% (from 0.333 g to 0.357 g) without a significant difference.

In the cases of cell density, the similar tendency was observed as that of the dry cell weight. The cell density of the biofilm (detected by resuspending) reduced by 37.0% from 4.091 × 10^5^ ml^−1^ in the control to 2.577 × 10^5^ ml^−1^ in the treatment, while the cell density in culture suspension increased by 6.7% from 5.466 × 10^5^ ml^−1^ to 5.833 × 10^5^ ml^−1^ without a significant difference. The decreases in the sum of cell dry weight and cell density in the treatment with bacterial supernatant crude extract were contributed by the significant reduction in the biofilm fraction.

In the whole culture of diatom, 42% of diatom cells (in terms of cell dry weight) formed the biofilm in the control. Treatment with supernatant crude extract from the tested strain significantly decreased the percentage of biofilm cells to 29%, whereas the percentage of floating cells increased from 58% to 71%. Accordingly, the ratio of biofilm cells/floating cells decreased from 0.736 to 0.414. For data described by cell density, the consistent results occurred. Due to the good correlation between dry cell weight and cell density, the subsequent results were expressed by dry cell weight only.

### 3.5. Effect of the Bacterial Supernatant Crude Extract on EPS Production of Growing Diatom

The EPS of diatom *Stauroneis* sp. grown in the presence of the supernatant crude extract of the tested strain were fractionated and measured. The treatment of supernatant crude extract led to significant increase in the EPS dry weight (Table 2) with minor reduction in total biomass (Table 1). Among different EPS fractions, the treatment led to a higher proportion of SL-EPS (from 74.00% to 91.29%) in the EPS distribution. Taking the slight variance of the biomass into account, the EPS production per diatom cell dry weight was 16.66 (g/g cell dry weight) in the control and increased to 41.59 (g/g cell dry weight) with treatment (2.49-fold) (Table 2). The supernatant crude extract led the EPS production per diatom biomass to increase, especially for SL-EPS (3.08-fold higher than control). The production of BF-TB-EPS per biomass also significantly increased to 2.71 (g/g cell dry weight), which was 1.76-fold higher than that of 1.54 (g/g cell dry weight) in the control.

### 3.6. Effect of the Bacterial Supernatant Crude Extract on the Monosaccharide Compositions of Diatom EPS Fractions

**Figure 5:**
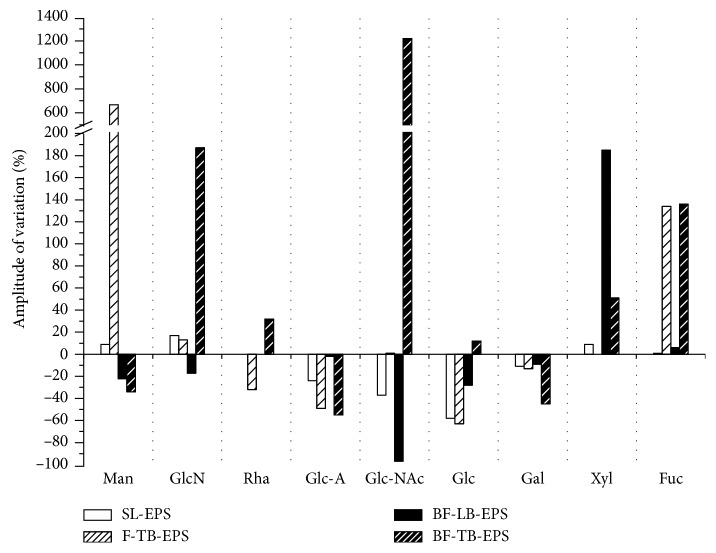
Variation amplitude for monosaccharides in EPS of diatom *Stauroneis* sp. treated by supernatant crude extract of *Psychrobacter* sp. SL-EPS and F-TB-EPS were prepared from the suspension phase of the treated diatom *Stauroneis* sp. culture, and BF-LB-EPS and BF-TB-EPS were prepared from the biofilm phase. Based on the HPLC profiles of samples and standard monosaccharides, the correction factors (*f*_1/2_), molar ratios (*R*_1/2_), and mole percentages were calculated. The amplitude of variation (%) for each monosaccharide in an EPS fraction was calculated as the variation (mole percentages) between the treatment and control divided by the mole percentage in the control.

The EPS fractions of *Stauroneis* sp. were hydrolysed and subjected to HPLC analysis. The results of the control and treatment (growing in the presence of the supernatant crude extract of tested strain) are shown in Table 3 and Figure 5, respectively.

As shown in Table 3, the EPS of the untreated *Stauroneis* sp. included nine monosaccharides of Man, GlcN, Rha, Glc-A, Glc-NAc, Glc, Gal, Xyl, and Fuc, which were identified by comparison to standards. The soluble EPS fraction and other fractions showed qualitatively similar monosaccharides compositions. The major monosaccharide in the SL-EPS fraction was Xyl, with significant levels of Gal, Man, Glc-A, and GlcN and slight levels of Fuc, Glc, and Glc-NAc. The dominant monosaccharide in each fraction varied: for both F-TB-EPS and BF-TB-EPS, Glc was the dominant monosaccharide, with mole percentages of 37.9% and 61.0%, respectively. Xyl was the most abundant monomer of SL-EPS, with a mole percentage of 37.0%, while Man was most abundant in BF-LB-EPS with a mole percentage of 23.2%.

As shown in Figure 5, treatment with the supernatant crude extract from the tested strain led to altered levels of all monosaccharides. Among the four fractions, treatment with the supernatant crude extract caused the largest changes in BF-TB-EPS and the smallest in SL-EPS. In the nine monomers detected, only four monomers altered in the same direction in all four EPS fractions: Glc-A and Gal always decreased, and Xyl and Fuc increased in all EPS fractions. Glc-NAc exhibited the largest increase (1219%) in BF-TB-EPS, followed by Man (667%) in F-TB-EPS. The changes in other monomers were less than 200%.

## 4. Discussion

In our previous study, several strains with remarkable activity against diatom biofilm formation of *Amphora* sp., *Nitzschia closterium*, *Sellaphora* sp., and *Stauroneis* sp. were screened from a sponge-associated bacterial bank [29]. In extension screening, the UST050418-708 strain was found and identified as *Psychrobacter* sp. in this study (Figure 2). Its activity against diatom biofilm formation was confirmed to be higher than those of most strains in the previous study [29], with an inhibition ratio of >90% against all four tested diatom species of *Amphora* sp., *Nitzschia closterium*, *Nitzschia frustulum*, and *Stauroneis* sp. in Figure 3.

In natural habitats, microphytobenthic (MPB) biofilms are widespread and are mainly composed of diatoms and bacteria [5]. Inside these biofilms, multiple interactions exist between MPB and bacteria, including trophic pathways and other potential interactions, including competition for nutrients and negative cell/cell interactions [4]. Understanding whether trophic competition between bacteria and diatoms is important for activities against diatom biofilm formation, the culture of tested strain was divided into cells and the supernatant to investigate their effect on diatom biofilm formation separately. The results in Figure 4 show that the supernatant was significantly more effective than cells against diatom biofilm formation, and that the competition for nutrients did not significantly contribute to the inhibition effect of the tested strain against biofilm formation of *Stauroneis* sp. Our results were similar to many reports of the cell-free supernatant, such as the supernatant of *Pseudomonas fluorescens* containing the quorum sensing signal affecting the growth, biofilm development, and spoilage potential of *Shewanella baltica* [42], the cell-free supernatant of a marine bacterium *Pseudoalteromonas haloplanktis* containing a signal molecule that identifies as a long-chain fatty acid active against *Staphylococcus epidermidis* [43, 44], and the spent medium of a coisolated bacteria inducing diatom *Achnanthidium minutissimum* capsule and biofilm formation [10]. Therefore, we propose that metabolites of the tested strain in the supernatant are responsible for the activity.

The results in Table 1 indicate that the extract from the culture supernatant of the tested strain significantly reduces the biomass of diatoms which formed biofilm in the culture and did not change the floating biomass significantly. The stable biomass in the floating phase indicates no significant lethal effect of the crude extract. The decreased biomass in the biofilm phase proves that the extract made the cells difficult to form biofilm and to grow to high density. The treatment did significantly alter the distribution of planktonic versus biofilm cells. The significant changes in the cell distribution proved that the supernatant of the tested strain possessed high activity against diatom biofilm formation rather than lethal effect [18, 19].

We were interested in how the supernatant crude extract led to the changes in the EPS fractions. Diatom cells in the MPB biofilms secrete a wide range of EPS, which are major components of the biofilm matrix [4–6]. These EPS have been described as regulators of bacterial development [5, 45]. Therefore, the EPS is absolutely necessary for biofilm formation and plays important functions in the interactions of MPB and bacteria. Algal EPS production is considered being regulated by environmental factors [11, 12, 46]. The responses in the EPS production are assumed to be an attempt of diatom to adapt to environmental changes [47–49]. In the present study, the treatment of supernatant crude extract of the tested strain led diatom *Stauroneis* sp. to produce 2-fold more total EPS, both in terms of total EPS weight and cell quota, as shown in Table 2. The increase in EPS production indicates that the supernatant crude extract of the tested strain made diatoms difficult to form biofilm, and that the treated diatom was struggling to complete biofilm formation by producing more total EPS.

Besides the responses in the total EPS production of the treated diatom being observed, further investigation on the responses of various fractions of EPS was also carried out. The EPS of diatoms can be classified in two main fractions: one of which is colloidal EPS that are soluble in saline water and excreted in the vicinity of cells, and the other of which is the bound EPS that is tightly attached to the algal cell wall. Bound EPS may be involved in the cell-cell communication of the bacteria-diatom consortium in addition to having adhering properties; such communication is expected to contribute to biofilm development and surface colonization [5]. In a previous study, the diatom *Achnanthidium minutissimum*, which normally does not form biofilm and in which the cells grow completely suspended, was induced to form biofilm in the presence of a coisolated bacteria [10]. The experiments following the changes of different fractions of the diatom EPS found stable total amount with reducing dissolved and increasing insoluble EPS [10]. In our investigation of responses in various fractions of EPS (Table 2), the increase in EPS production (g/g cell dry weight) was mainly contributed by SL-EPS and BF-TB-EPS in the treatment. SL-EPS is produced by both biofilm cells and floating cells in the culture. BF-TB-EPS should be the key fraction for biofilm formation [10]. It appears that treated diatom cells must produce more BF-TB-EPS to complete biofilm formation than untreated cells. Moreover, the increased production of BF-TB-EPS suggests that the treated EPS exhibited lower efficiency to embed diatom cells onto the substrate surface to form biofilm compared to those from the control diatom.

To understand low efficiency of treated EPS in biofilm formation, the monomeric composition of EPS was studied. The treatment of supernatant crude extract of the tested strain effected remarkable changes on the EPS monomeric compositions of the diatom *Stauroneis* sp. As shown in Figure 5, the EPS of treated diatom contained less Glc-A and Gal and more Xyl and Fuc in all of the EPS fractions compared to the control. The content of Glc-NAc increased with the largest amplitude of variation by 1219% in BF-TB-EPS. There are reports which proposed that surface-active polysaccharides, such as acidic sugars, including uronic acids and sulfonic sugars, were correlated with the coagulation efficiency [50]. It was also reported that more than 90% of the EPS fraction being composed of different acidic polysaccharides led to the strong adhesive nature of *Amphora* sp. [38]. Therefore, the reduced content of acidic sugars, such as Glc-A, and increased content of alkaline sugars, such as Glc-NAC, in the EPS of diatom *Stauroneis* sp. might be important for the low efficiency of treated EPS and the activity of the supernatant crude extract from the tested strain against diatom biofilm formation.

The active strain was identified as *Psychrobacter* sp., a genus with many reported characteristics, including cold and salt tolerance, and a unique cellular fatty acid content [51, 52]. The activity against diatom biofilm formation of *Psychrobacter* species is reported here for the first time. Further studies to isolate the active metabolites produced by this strain should lead to the discovery of new active compounds against diatom biofilm formation.

In conclusion, a sponge-associated bacteria strain UST050418-708, which was identified as *Psychrobacter* sp. and found sharing very high 16S rDNA sequence similarities with *Psychrobacter glacincola* in this study, possesses remarkable activities against biofilm formation of different species of benthic diatoms. The activity for this strain was found in the culture supernatant. The crude extract of the supernatant altered cell distribution of diatom *Stauroneis* sp. such that fewer cells formed biofilms. Importantly, the supernatant crude extract of the tested *Psychrobacter* strain caused significant changes not only in the productions of BF-TB-EPS and SL-EPS but also in monosaccharide composition of the diatom *Stauroneis* sp, especially a decrease in Glc-A of all EPS fractions and an increase in Glc-NAC of BF-TB-EPS. Metabolites of this strain are proposed as a promising source for novel active compounds against diatom biofilm formation.

## Figures and Tables

**Table 1:** Cell distributions in the floating phase and biofilm of diatom *Stauroneis* sp. incubated in the presence of bacterial supernatant crude extract.

Cell distribution	Control			Treatment		
	Dry weight (g)	Cell density (×10^5^ ml^−1^)	Percentage (%)	Dry weight (g)	Cell density (×10^5^ ml^−1^)	Percentage (%)
Biofilm	0.245 ± 0.014	4.091 ± 0.218	42	0.147 ± 0.016^*∗∗*^	2.577 ± 0.255^*∗∗*^	29
Floating	0.333 ± 0.013	5.466 ± 0.199	58	0.357 ± 0.021	5.833 ± 0.326	71
Sum	0.578 ± 0.008	9.557 ± 0.124	—	0.504 ± 0.010^*∗∗*^	8.409 ± 0.157^*∗∗*^	—
Biofilm/floating	0.736 ± 0.066	0.750 ± 0.063	—	0.414 ± 0.070^*∗∗*^	0.444 ± 0.068^*∗∗*^	—

Cell weights and cell densities were independently measured at least three times, and the means and standard deviations are shown. Independent *t*-test was used to compare the control and treatment. Percentages were calculated based on the means of cell weights and indicated the cell distribution between biofilm and floating phases. ^*∗∗*^*P* < 0.01.

**Table 2:** EPS production per biomass of diatom *Stauroneis* sp. incubated in the presence of bacterial supernatant crude extract.

Fraction	Control					Treatment				
	SL-EPS	F-TB-EPS	BF-LB-EPS	BF-TB-EPS	Total	SL-EPS	F-TB-EPS	BF-LB-EPS	BF-TB-EPS	Total
EPS dry weight (g)	7.13 ± 0.01	0.33 ± 0.05	1.79 ± 0.01	0.38 ± 0.02	9.63 ± 0.03	19.13 ± 0.01^*∗∗*^	0.39 ± 0.05	1.04 ± 0.02^*∗∗*^	0.40 ± 0.02	20.96 ± 0.03^*∗∗*^
Percentage (%)	74.00	3.46	18.62	3.92	100	91.29	1.87	4.94	1.90	100
EPS production (g/g cell dry weight)	12.33 ± 0.36	1.00 ± 0.13	7.34 ± 0.48	1.54 ± 0.04	16.66 ± 0.50	37.96 ± 0.27^*∗∗*^	1.10 ± 0.12	7.05 ± 0.04	2.71 ± 0.14^*∗∗*^	41.59 ± 0.24^*∗∗*^

Dry weights of fractions were independently measured at least three times, and the means and standard deviations are shown. Percentages were calculated based on the means of dry weights. EPS production was calculated as the EPS weight divided by cell dry weight, and means and standard deviations are shown. Independent *t*-test was used to compare the control and treatment. ^*∗∗*^*P* < 0.01.

**Table 3:** Monosaccharide composition for EPS fractions prepared from untreated diatom *Stauroneis* sp.

Fractions	Monosaccharides (mol%)								
	Man	GlcN	Rha	Glc-A	Glc-NAc	Glc	Gal	Xyl	Fuc
SL-EPS	14.8	10.4	0	12.2	0.8	2.9	15.5	37.0	6.6
F-TB-EPS	4.0	7.9	13.0	10.9	1.1	37.9	24.1	0	1.2
BF-LB-EPS	23.2	16.1	0	2.4	11.7	13.6	13.9	13.0	6.1
BF-TB-EPS	17.8	1.9	0.6	1.9	0.1	61.0	14.9	0.7	0.9

Mole percentage (mol%) of each monosaccharide was calculated on the basis of HPLC profiles of each fractions, and data represent the average of duplicate experiments.

## References

[1] Mol V. P. L., Raveendran T. V., Parameswaran P. S. (2009). Antifouling activity exhibited by secondary metabolites of the marine sponge, *Haliclona exigua* (Kirkpatrick). *International Biodeterioration and Biodegradation*.

[2] Schultz M. P. (2007). Effect of coating roughness and biofouling on ship resistance and powering. *Biofouling*.

[3] Mejdandžić M., Ivanković T., Pfannkuchen M. (2015). Colonization of diatoms and bacteria on artificial substrates in the northeastern coastal Adriatic Sea. *Acta Botanica Croatica*.

[4] Agogué H., Mallet C., Orvain F., Crignis M. D., Mornet F., Dupuy C. (2014). Bacterial dynamics in a microphytobenthic biofilm: a tidal mesocosm approach. *Journal of Sea Research*.

[5] Orvain F., Crignis M. D., Guizien K., Lefebvre S., Mallet C., Takahashi E. (2014). Tidal and seasonal effects on the short-term temporal patterns of bacteria, microphytobenthos and exopolymers in natural intertidal biofilms (Brouage, France). *Journal of Sea Research*.

[6] Underwood G. J. C., Paterson D. M. (2003). The importance of extracellular carbohydrate production by marine epipelic diatoms. *Advances in Botanical Research*.

[7] Vanelslander B., Paul C., Grueneberg J. (2012). Daily bursts of biogenic cyanogen bromide (BrCN) control biofilm formation around a marine benthic diatom. *Proceedings of the National Academy of Sciences of the United States of America*.

[8] Cao S., Wang J., Li D., Chen D. (2013). Ecological and social modeling for migration and adhesion pattern of a benthic diatom. *Ecological Modelling*.

[9] Kumar V., Rao D., Thomas T., Kjelleberg S., Egan S. (2010). Antidiatom and antibacterial activity of epiphytic bacteria isolated from *Ulva lactuca* in tropical waters. *World Journal of Microbiology and Biotechnology*.

[10] Windler M., Leinweber K., Bartulos C. R., Philipp B., Kroth P. G. (2015). Biofilm and capsule formation of the diatom *Achnanthidium minutissimum* are affected by a bacterium. *Journal of Phycology*.

[11] Pierre G., Graber M., Rafiliposon B. A. (2012). Biochemical composition and changes of extracellular polysaccharides (ECPS) produced during microphytobenthic biofilm development (Marennes-Oléron, France). *Microbial Ecology*.

[12] Pierre G., Zhao J. M., Orvain F., Dupuy C., Klein G. L., Graber M. (2014). Seasonal dynamics of extracellular polymeric substances (EPS) in surface sediments of a diatom-dominated intertidal mudflat (Marennes–Oléron, France). *Journal of Sea Research*.

[13] Bennke C. M., Neu T. R., Fuchs B. M., Amann R. (2013). Mapping glycoconjugate-mediated interactions of marine Bacteroidetes with diatoms. *Systematic and Applied Microbiology*.

[14] Verneuil L., Silvestre J., Randrianjatovo I., Marcato-Romain C. E., Girbal-Neuhauser E., Mouchet F. (2015). Double walled carbon nanotubes promote the overproduction of extracellular protein-like polymers in *Nitzschia palea*: an adhesive response for an adaptive issue. *Carbon*.

[15] Decho A. W. (2000). Microbial biofilms in intertidal systems: an overview. *Continental Shelf Research*.

[16] Higgins M. J., Molino P., Mulvaney P., Wetherbee R. (2003). The structure and nanomechanical properties of the adhesive mucilage that mediates diatom-substratum adhesion and motility. *Journal of Phycology*.

[17] Wustman B. A., Gretz M. R., Hoagland K. D. (1997). Extracellular matrix assembly in diatoms (Bacillariophyceae) (Ι. A model of adhesives based on chemical characterization and localization of polysaccharides from the marine diatom *Achnanthes longipes* and other diatoms). *Plant Physiology*.

[18] Qian P. Y., Xu Y., Fusetani N. (2009). Natural products as antifouling compounds: recent progress and future perspectives. *Biofouling*.

[19] Qian P. Y., Li Z. R., Xu Y., Li Y. X., Fusetani N. (2015). Mini-review: marine natural products and their synthetic analogs as antifouling compounds: 2009–2014. *Biofouling*.

[20] Taylor M. W., Radax R., Steger D., Wagner M. (2007). Sponge-associated microorganisms: evolution, ecology, and biotechnological potential. *Microbiology and Molecular Biology Reviews*.

[21] Dash S., Jin C. L., Lee O. O., Xu Y., Qian P. Y. (2009). Antibacterial and antilarval-settlement potential and metabolite profiles of novel sponge-associated marine bacteria. *Journal of Industrial Microbiology and Biotechnology*.

[22] Dash S., Nogata Y., Zhou X. J. (2011). Poly-ethers from *Winogradskyella poriferorum*: antifouling potential, time-course study of production and natural abundance. *Bioresource Technology*.

[23] Dobretsov S. V., Qian P. Y. (2002). Effect of bacteria from surface of the green seaweed *Ulva reticulata* on marine micro- and macrofouling. *Biofouling*.

[24] Fusetani N. (2004). Biofouling and antifouling. *Nature Products Reports*.

[25] Kennedy J., Baker P., Piper C. (2009). Isolation and analysis of bacteria with antimicrobial activities from the marine sponge *Haliclona simulans* collected from Irish waters. *Marine Biotechnology*.

[26] Lee O. O., Qian P. Y. (2003). The chemical control of bacterial epiosis and larval settlement of *Hydroides elegans* in the red sponge *Mycale adherens*. *Biofouling*.

[27] Santos O. C. S., Pontes P. V. M. L., Santos J. F. M., Muricy G., Giambiagi-deMarval M., Laport M. S. (2010). Isolation, characterization and phylogeny of sponge-associated bacteria with antimicrobial activities from Brazil. *Research in Microbiology*.

[28] Thiel V., Imhoff J. F. (2003). Phylogenetic identification of bacteria with antimicrobial activities isolated from Mediterranean sponges. *Biomolecular Engineering*.

[29] Jin C. L., Xin X. Y., Yu S. Y. (2014). Antidiatom activity of marine bacteria associated with sponges from San Juan Island, Washington. *World Journal of Microbiology and Biotechnology*.

[30] Xin X. Y., Huang G. H., Zhou X. J. (2017). Potential antifouling compounds with antidiatom adhesion activities from the sponge-associated bacteria, *Bacillus pumilus*. *Journal of Adhesion Science and Technology*.

[31] Lind J. L., Heimann K., Miller E. A., van Vliet C., Hoogenraad N. J., Wetherbee R. (1997). Substratum adhesion and gliding in a diatom are mediated by extracellular proteoglycans. *Planta*.

[32] Mcconville M. J., Wetherbee R., Bacic A. (1999). Subcellular location and composition of the wall and secreted extracellular sulphated polysaccharides/proteoglycans of the diatom *Stauroneis amphioxys* Gregory. *Protoplasma*.

[33] Lee O. O., Wong Y. H., Qian P. Y. (2009). Inter- and intraspecific variations of bacterial communities associated with marine sponges from San Juan Island, Washington. *Applied and Environmental Microbiology*.

[34] Li H., Sun H., Bai X. (2016). HC2 of *Pseudomonas* sp. induced enteritis in *Hippocampus japonicas*. *Aquaculture Research*.

[35] Guillard R. R. L., Ryther J. H. (1962). Studies of marine planktonic diatoms. I. *Cyclotella nana* Hustedt and *Detonula confervacea* Cleve. *Canadian Journal of Microbiology*.

[36] Leflaive J., Ten-Hage L. (2011). Impairment of benthic diatom adhesion and photosynthetic activity by 2E,4E-decadienal. *Research in Microbiology*.

[37] Xu H. C., Cai H. Y., Yu G. H., Jiang H. L. (2013). Insights into extracellular polymeric substances of cyanobacterium *Microcystis aeruginosa* using fractionation procedure and parallel factor analysis. *Water Research*.

[38] Zhang S. J., Xu C., Santschi P. H. (2008). Chemical composition and 234Th (IV) binding of extracellular polymeric substances (EPS) produced by the marine diatom *Amphora* sp.. *Marine Chemistry*.

[39] Liang J. R., Ai X. X., Gao Y. H., Chen C. P. (2013). MALDI-TOF MS analysis of the extracellular polysaccharides released by the diatom *Thalassiosira pseudonana*. *Journal of Applied Phycology*.

[40] Wang H. X., Zhao J., Li D. M. (2015). Comparison of polysaccharides of *Haliotis discus* hannai and *Volutharpa ampullaceal* perryi by PMP-HPLC-MS^n^ analysis upon acid hydrolysis. *Carbohydrate Research*.

[41] Yang X., Zhao Y., Wang Q., Wang H., Mei Q. (2005). Analysis of the monosaccharide components in *Angelica* polysaccharides by high performance liquid chromatography. *Analytical Sciences*.

[42] Zhao A., Zhu J., Ye X., Ge Y., Li J. (2016). Inhibition of biofilm development and spoilage potential of *Shewanella baltica* by quorum sensing signal in cell-free supernatant from *Pseudomonas fluorescens*. *International Journal of Food Microbiology*.

[43] Casillo A., Papa R., Ricciardelli A. (2017). Anti-biofilm activity of a long-chain fatty aldehyde from Antarctic *Pseudoalteromonas haloplanktis* TAC125 against *Staphylococcus epidermidis* biofilm. *Frontiers in Cellular and Infection Microbiology*.

[44] Parrilli E., Papa R., Carillo S. (2015). Anti-biofilm activity of *Pseudoalteromonas haloplanktis* tac125 against *Staphylococcus epidermidis* biofilm: evidence of a signal molecule involvement. *International Journal of Immunopathology and Pharmacology*.

[45] Lubarsky H. V., Hubas C., Chocholek M. (2010). The stabilisation potential of individual and mixed assemblages of natural bacteria and microalgae. *PLoS One*.

[46] Pletikapić G., Žutić V., Vinković Vrček I., Svetličić V. (2012). Atomic force microscopy characterization of silver nanoparticles interactions with marine diatom cells and extracellular polymeric substance. *Journal of Molecular Recognition*.

[47] Ai X. X., Liang J. R., Gao Y. H. (2015). MALDI-TOF MS analysis of the extracellular polysaccharides released by the diatom *Thalassiosira pseudonana* under various nutrient conditions. *Journal of Applied Phycology*.

[48] Aslam S. N., Tania C., Thomas D. N., Underwood G. J. C. (2012). Production and characterization of the intra- and extracellular carbohydrates and polymeric substances (EPS) of three sea-ice diatom species, and evidence for a cryoprotective role for EPS. *Journal of Phycology*.

[49] Gügi B., Le C. T., Burel C., Lerouge P., Helbert W., Bardor M. (2015). Diatom-specific oligosaccharide and polysaccharide structures help to unravel biosynthetic capabilities in diatoms. *Marine Drugs*.

[50] Chow J. S., Lee C., Engel A. (2015). The influence of extracellular polysaccharides, growth rate, and free coccoliths on the coagulation efficiency of *Emiliania huxleyi*. *Marine Chemistry*.

[51] Barney B. M., Wahlen B. D., Garner E., Wei J., Seefeldt L. C. (2012). Differences in substrate specificities of five bacterial wax ester synthases. *Applied and Environmental Microbiology*.

[52] Chain P. S., Grzymski J. J., Ponder M. A., Ivanova N., Bergholz P. W., Bartolo G. D. (2010). The genome sequence of *Psychrobacter arcticus* 273-4, a psychroactive *Siberian permafrost* bacterium, reveals mechanisms for adaptation to low-temperature growth. *Applied and Environmental Microbiology*.

